# Identification of the angiogenesis related genes for predicting prognosis of patients with gastric cancer

**DOI:** 10.1186/s12876-021-01734-4

**Published:** 2021-04-01

**Authors:** Sheng Zheng, Zizhen Zhang, Ning Ding, Jiawei Sun, Yifeng Lin, Jingyu Chen, Jing Zhong, Liming Shao, Zhenghua Lin, Meng Xue

**Affiliations:** 1 Department of Gastroenterology, The Second Affiliated Hospital of Zhejiang University School of Medicine, 88 Jiefang Road, Hangzhou, 310009 Zhejiang China; 2 Institute of Gastroenterology, Zhejiang University, Hangzhou, China

**Keywords:** Gastric cancer, Angiogenesis, Prognostic, Gene

## Abstract

**Introduction:**

Angiogenesis is a key factor in promoting tumor growth, invasion and metastasis. In this study we aimed to investigate the prognostic value of angiogenesis-related genes (ARGs) in gastric cancer (GC).

**Methods:**

mRNA sequencing data with clinical information of GC were downloaded from The Cancer Genome Atlas (TCGA) and the Gene Expression Omnibus (GEO) databases. The differentially expressed ARGs between normal and tumor tissues were analyzed by limma package, and then prognosis‑associated genes were screened using Cox regression analysis. Nine angiogenesis genes were identified as crucially related to the overall survival (OS) of patients through least absolute shrinkage and selection operator (LASSO) regression. The prognostic model and corresponding nomograms were establish based on 9 ARGs and verified in in both TCGA and GEO GC cohorts respectively.

**Results:**

Eighty-five differentially expressed ARGs and their enriched pathways were confirmed. Significant enrichment analysis revealed that ARGs-related signaling pathway genes were highly related to tumor angiogenesis development. Kaplan–Meier analysis revealed that patients in the high-risk group had worse OS rates compared with the low-risk group in training cohort and validation cohort. In addition, RS had a good prognostic effect on GC patients with different clinical features, especially those with advanced GC. Besides, the calibration curves verified fine concordance between the nomogram prediction model and actual observation.

**Conclusions:**

We developed a nine gene signature related to the angiogenesis that can predict overall survival for GC. It’s assumed to be a valuable prognosis model with high efficiency, providing new perspectives in targeted therapy.

**Supplementary Information:**

The online version contains supplementary material available at 10.1186/s12876-021-01734-4.

## Introduction

Gastric cancer (GC) is a common gastrointestinal malignancy which is the fifth most frequently diagnosed cancer (1,000,000 case/year) and the third leading cause of cancer-related deaths (783,000 case/year) toll worldwide [1]. Even if diagnostic and therapeutic strategies have been improved over the past 20 years, the outcome is still poor with overall 5-year survival rate less than 40% [2–4]. The discovery and application of molecular biomarkers have improved the prognosis evaluation and recognition of potential high-risk GC patients [5]. Therefore, it is of great significance to clarify the interactions of key molecules during the occurrence and development of GC, which will help to prevent GC and find new therapeutic targets.

Angiogenesis is the formation of new blood vessels from pre-existing ones through a process called germination [6]. It is mainly involved in embryonic development and wound healing under physiological conditions [7]. Without vascular supply, the tumors cannot grow over 1–2 mm, so pathological angiogenesis is one of the hallmarks of the tumor [8, 9]. In order to support the high proliferation rate and high metabolic rate of cancer cells, it’s required to develop new vascular networks rapidly, which is driven by angiogenic factors [10]. These neonatal blood vessels not only provide the necessary oxygen and nutrients for maintaining the rapid growth and proliferation of tumor cells, but also provide the possibility for tumor cells to enter the circulatory system and metastasize to the distance [11].

The expression pattern of angiogenesis factors was closely related to the prognosis of GC patients and has been studied as a therapeutic target [12]. For example, Ramucirumab (an VEGFR-2 antibody) can significantly prolonged overall survival (OS) of GC patients [13]. It has been approved by the Food and Drug Administration (FDA) as a second-line treatment for advanced GC in 2014. However, only few angiogenic factors have been determined to be associated with the prognosis of GC patients. Besides, most studies focused on the effect of a single gene on the prognosis, such as VEGF [14]. It will be more effective to identify the prognosis of GC by combining the expression levels of various angiogenesis factors.

In the present study, the clinical data of 375 GC patients were downloaded from The Cancer Genome Atlas (TCGA), and the correlation between the expressions of angiogenesis-related genes (ARGs) were analyzed. Besides, risk score (RS) was calculated as an independent index for overall survival (OS) prognosis based on ARGs. These findings reveal some key AGRs in gastric cancer and have a certain guiding significance for the follow-up study of tumor angiogenesis. In addition, these results can also provide an effective risk score formula for predicting the prognosis and guiding the management of GC patients.

## Materials and methods

### Selection of angiogenesis-related genes

AGRs were retrieved from the GeneCards website (https://www.genecards.org/) with the term “angiogenesis”. Relevance scores was used to indicate the intensity of the correlation between genes and angiogenic activity, ranging from 0 to 100. A high score represents a strong correlation. The ARGs with correlation scores > 5 were screened for follow-up study and analysis.

### Acquisition of gastric cancer datasets

The original RNA-sequencing (RNA-seq) datasets and clinical characteristics of the TCGA gastric cancer cohort were downloaded from the TCGA website (https://portal.gdc.cancer.gov/). GSE84437, including 433 GC samples dataset, was obtained from the Gene Expression Omnibus (GEO, https://www.ncbi.nlm.nih.gov/geo/) for the validation study.

### Differentially expressed ARGs and enrichment analysis

The differentially expressed ARGs in the mRNA expression data of GC cohort were identified by the limma package in R software (FDR < 0.05, ∣logFC∣ > 1). Visualization (with volcano plots and heatmaps) was performed using the ggrepel, ggplot, and pheatmap packages in R software. The functional annotations of these ARGs by Gene Ontology (GO), including biological process, cellular component, and molecular function, were analyzed and visualized with the goplot package. Similarly, the KEGG pathway enrichment analysis was implemented from the KEGG pathway database (www.kegg.jp/kegg/kegg1.html).

### Protein–protein interaction network construction and module screening

The protein–protein interactions (PPIs) of all differentially expressed ARGs were identified using the STRING database (http://www.string-db.org/). The further constructed and visualized of the PPI network was used by Cytoscape 3.7.0 software. Subsequently, the important modules and genes were screened from the PPI network with scores > 5 and node counts > 5 by the MCODE (Molecular Complex Detection) plug-in.

### Establishing an individualized prognostic index according to ARGs

Combining mRNA expression levels of ARGs and clinical data, the differentially expressed ARGs with significant prognostic value were screened by univariate Cox regression analysis. Identified overall survival (OS) related genes were used to develop prognostic multiple-gene signatures. Least absolute shrinkage and selection operator (LASSO) Cox regression method was adopted to construct multivariable models with ARGs using the “glmnet” package for R software [15, 16]. In LASSO regression, only the genes with non-zero coefficient are selected to further calculate the risk score [17]. The best model is determined by maximizing the performance and using the least number of genes.

Subsequently, based on the linear combination of the expression level and the weighted regression coefficient obtained by LASSO Cox regression analysis, the prognostic risk score formula was established. Risk score (RS) = expression of gene 1 × β1 + expression of gene 2 × β2 + ⋯ + expression of gene n x βn. The median RS was chosen as a cutoff value to separately dichotomize TCGA-STAD cohorts into high-risk and low‐risk subgroups. Univariate and multivariate Cox regression analysis were used to evaluate the prognostic value of these genes in patients with GC. The survival curve was drawn by Kaplan–Meier (KM) method, and the difference of survival rate between high risk group and low risk group was evaluated by log-rank test. Receiver operating characteristic (ROC) analyses were performed in R “survival ROC”. Moreover, the clinical significance of these identified genes was evaluated. Then, these findings were tested in another GC cohort in GEO datasets through survival analysis and ROC curve analysis. Furthermore, the nomogram with calibration plots was built using rms R package to forecast the concordance between actual and predicted survival.

### Statistical analysis

All statistical analyses and plots were implemented through R software (version 3.6.0). The correlation between risk score and clinical characteristics was tested by X^2^ test. The Kaplan–Meier curve was drawn and the log-rank test was used to test the significant difference of OS among the groups. Univariate and multivariate Cox proportional hazard regression analyses were also used to evaluate the relationship between risk scores and OS. ROC analysis was used to detect the sensitivity and specificity of gene signature risk score in predicting survival. The area under ROC curve (AUC) can be used as an index of prognostic accuracy. In all analyses, *P* value < 0.05 was set to be statistically significant.

## Results

### Identification of differentially expressed ARGs

**Fig. 1:**
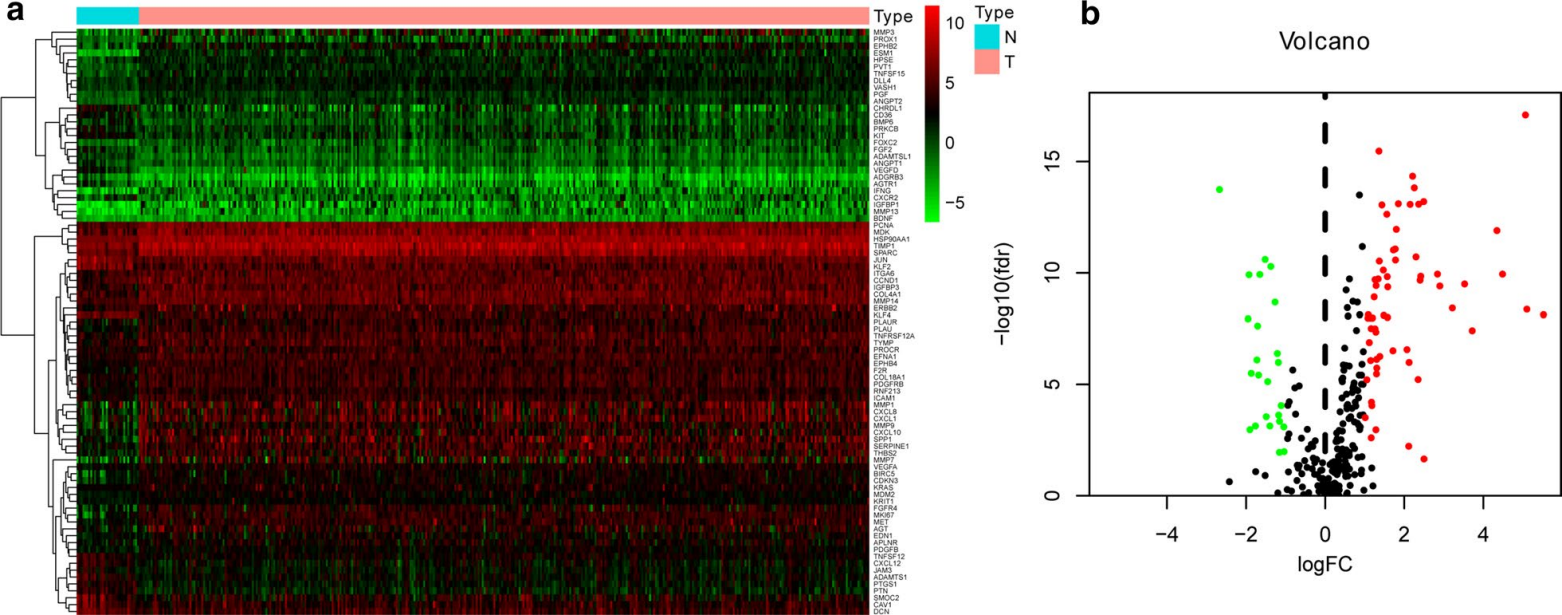
Differentially expressed ARGs between GC and normal gastric tissues. **a** The heatmap for the 338 ARGs from TCGA-STAD cohort; **b** volcano plot for screened ARGs

RNA-seq and clinical follow-up data were downloaded from TCGA-STAD dataset, including 375 gastric cancer samples and 32 normal samples. The demographic and clinical features of these patients were listed in Additional file 1: Table I. A total of 338 angiogenesis-related genes with relevance scores > 5 were acquired from GeneCards database. Then, we extracted and compared the expression level of 338 ARGs in RNA-seq data of normal samples and GC samples. The results indicated that there were 61 genes significantly upregulated and 24 genes significantly downregulated in GC (Fig. 1a, b).

### Biological functions and significant pathway analysis involved in the expression of ARGs

**Fig. 2:**
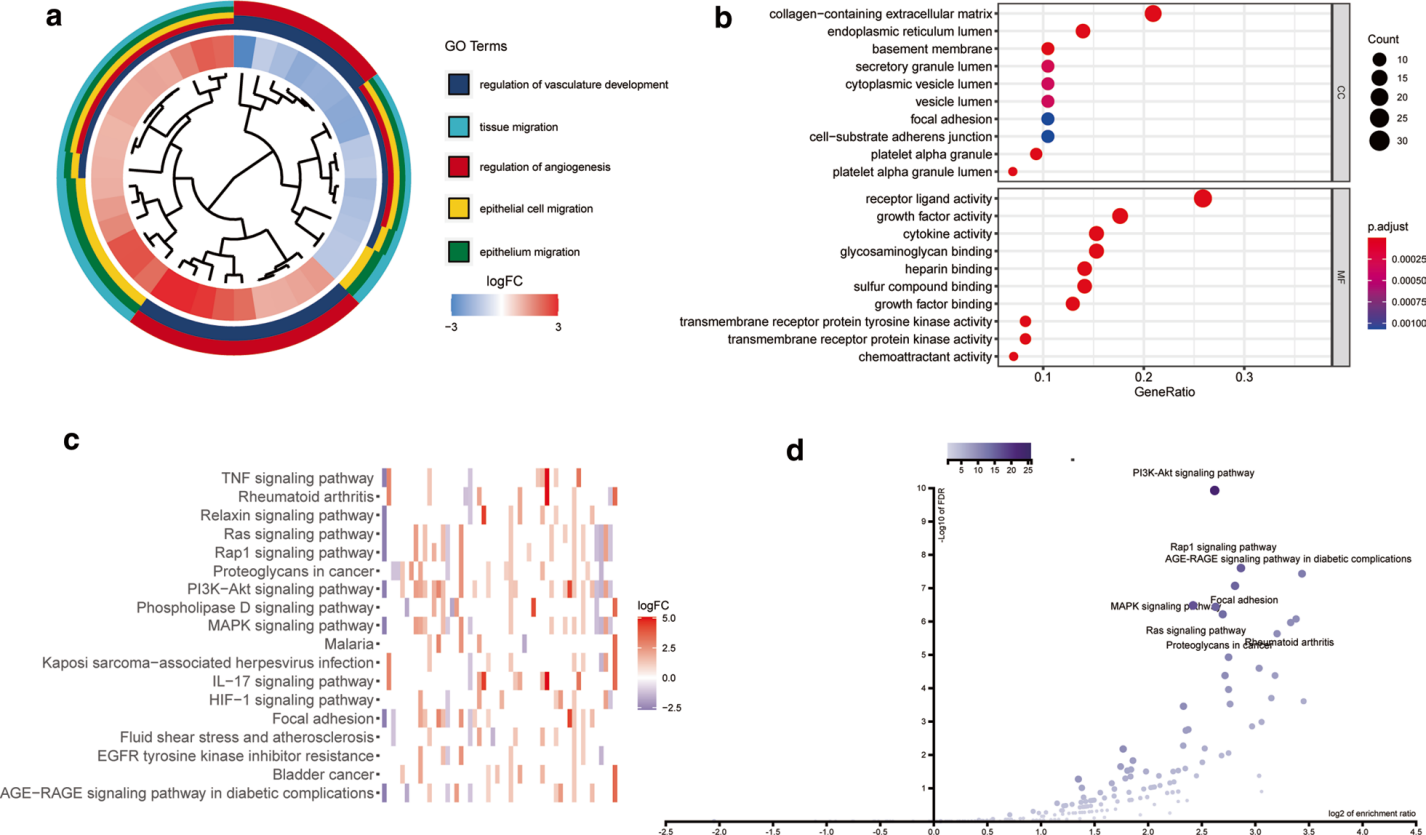
Functional enrichment analysis of differentially expressed ARGs. **a** Significantly enriched gene ontology (GO) terms of differentially expressed ARGs based on biological processes. **b** Significantly enriched of differentially expressed ARGs in GO terms based on cellular components and molecular functions. **c** The heatmap shows the LogFC values enriched by ARGs genes in different KEGG pathways. **d** Significantly enriched KEGG pathways of differentially expressed ARGs by Volcano

The biological functions and important pathways of 85 differentially expressed ARGs were analyzed. GO enrichment analysis showed that differential ARGs played an important role in angiogenesis and vasculature development in GC (Fig. 2a, b). The results of KEGG pathway enrichment analysis showed that the differentially expressed ARGs were mainly involved in the pathways related to tumor angiogenesis, including PI3K-Akt, MAPK and Rap1 pathways (Fig. 2c, d).

### Construction of PPI network and selection of key modules

**Fig. 3:**
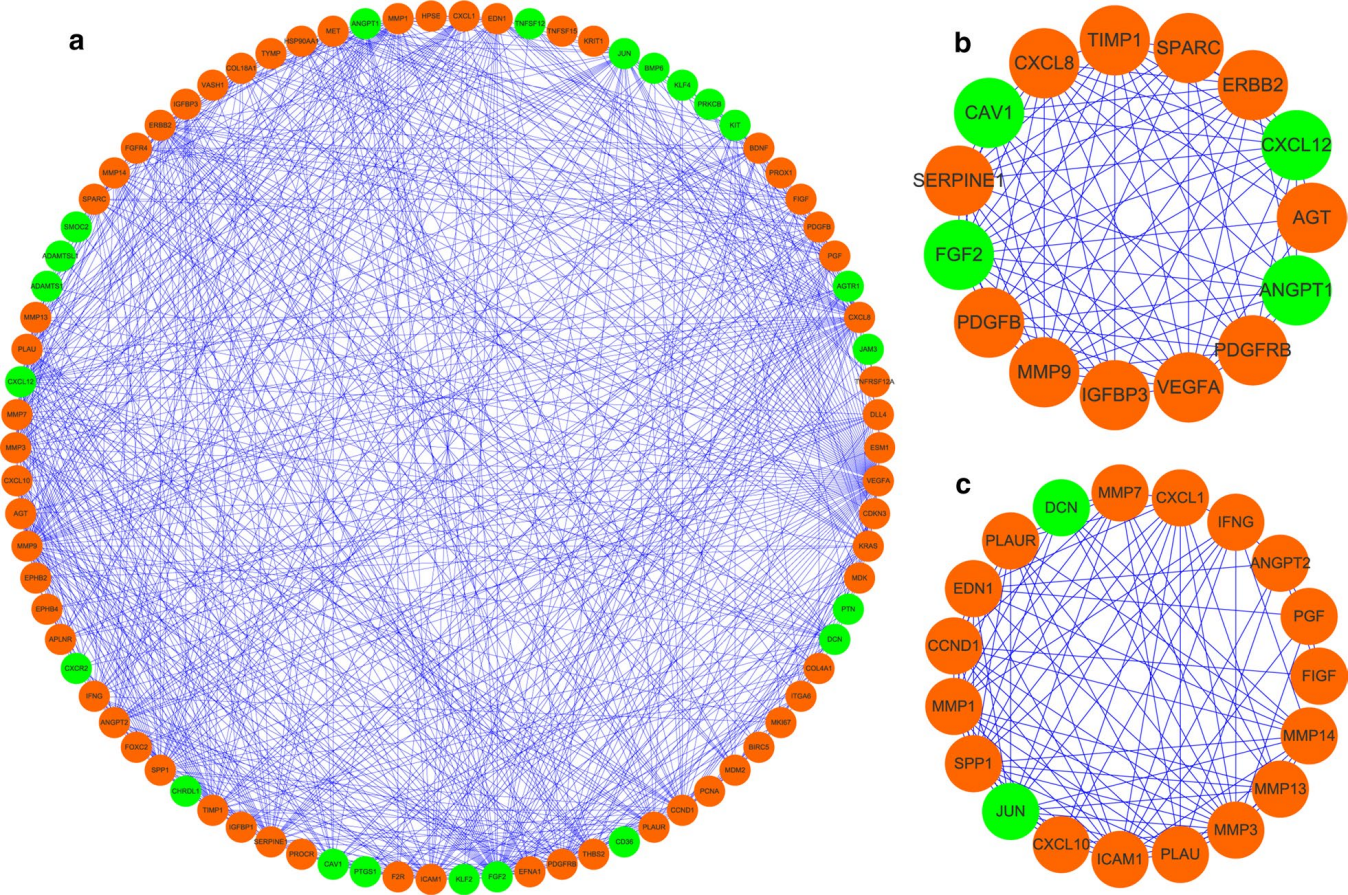
PPI network and module analysis. **a** The PPI network of all the differentially expressed ARGs visualized by Cytoscape. **b** Critical module 1 in PPI network. **c** Critical module 2 in PPI network

To further understand the potential molecular functions of differently expressed ARGs in STAD, we constructed the PPI network using Cytoscape software which incorporated 86 nodes and 752 edges based on the data from STRING database (Fig. 3a). Then, the co-expression network was further analyzed to detect potential critical modules via the MODE tool in Cytoscape. The top two significant modules, module 1 including 15 nodes and 83 edges (Fig. 3b), and module 2 consisting of 19 nodes and 97 edges were identified (Fig. 3c). The GO and pathway analyses showed that the genes from module 1 were mainly enriched in positive regulation of chemotaxis, epithelial cell migration, and vasculature development, whereas the genes in module 2 were significantly enriched in collagen catabolic process, extracellular matrix organization, leukocyte migration, and mechanical stimulus.

### Construction of an angiogenesis-related prognostic model

**Fig. 4:**
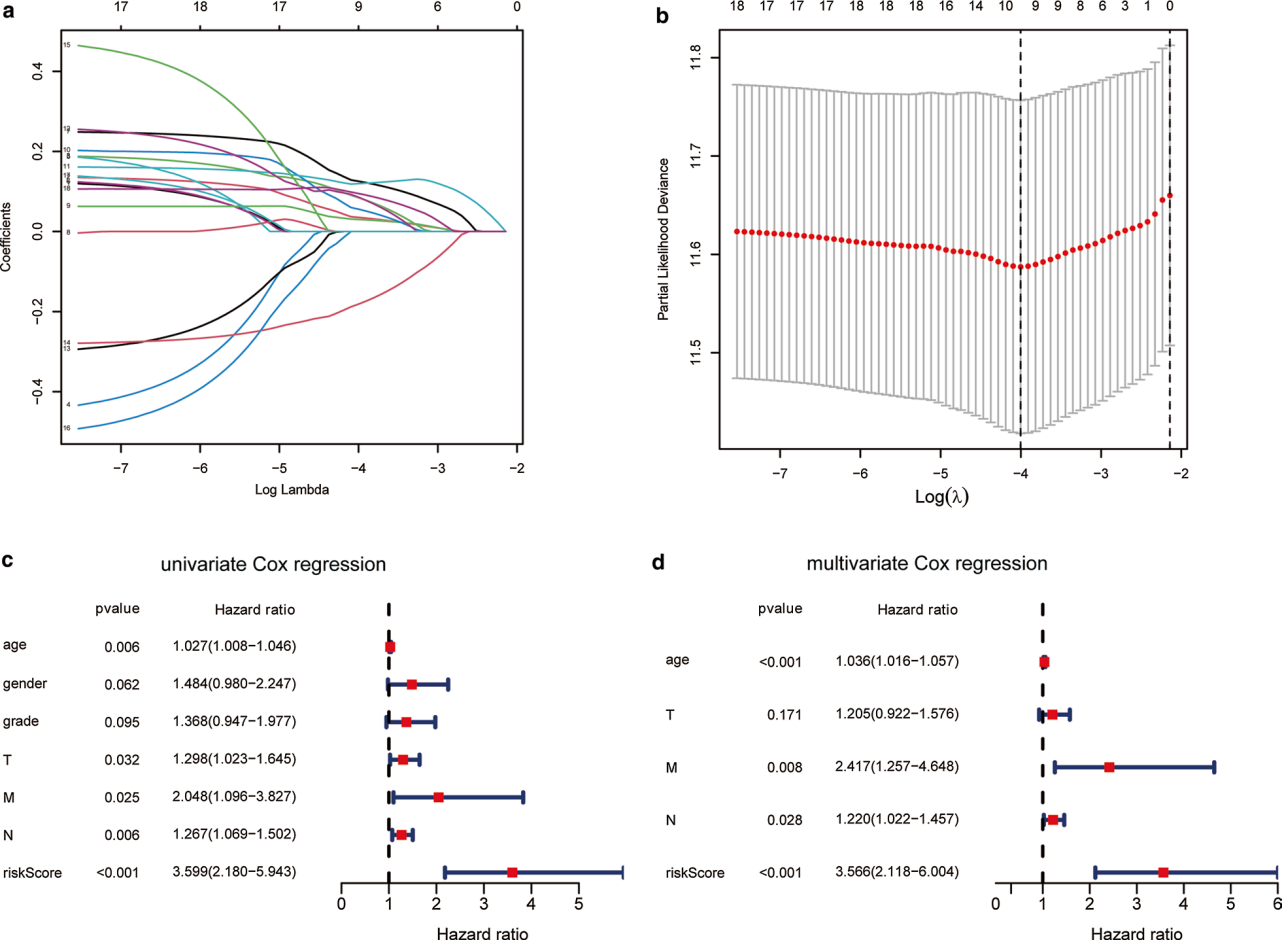
Establishment of ARGs prognostic model related to the prognosis of GC by lasso regression model. **a** LASSO coefficient profiles of the 18 ARGs. **b** A coefficient profile plot was generated against the log (lambda) sequence. **c** Univariate COX regression analysis for RS of GC patients in TCGA database. **d** Multivariate Cox regression analysis for RS of GC cancer patients in TCGA datasets

Univariate Cox regression analysis was used to analyze the correlation between the transcriptional expression of 85 differentially expressed ARGs and clinical data. It was found that 18 genes had significant associations (*P* < 0.05) with the prognosis of GC patients (Additional file 1: Table II). The LASSO COX regression analysis of 18 significant genes was implemented, and 9 genes (AGT, ANGPT1, SERPINE1, ANGPT2, PVT1, PROCR, KIT, PLAUR and CAV1) were screened out which could be the independent prognostic predictor in GC (Fig. 4a, b). The contribution rate of the 9 genes to the risk scoring model was weighted by the absolute value of the coefficient. According to the result of LASSO Cox regression analysis, the formula of the RS is as follows: RS = 0.035291 ∗ expression value of CAV1 + 0.084699 ∗ expression value of PLAUR + 0.125582 ∗ expression value of KIT + 0.030171 ∗ expression value of AGT + 0.055867 ∗ expression value of ANGPT1 + 0.119573 ∗ expression value of SERPINE1 + 0.085701 ∗ expression value of ANGPT2 + 0.094991 ∗ expression value of PROCR − 0.181397 ∗ expression value of PVT1.

In addition, univariate COX analysis and multivariate COX analysis were performed on the STAD-TCGA cohorts to further verify the reliability of RS in predicting the prognosis of patients with GC (Fig. 4c, d). The results showed that whether univariate COX regression analysis (HR 3.599, 95 % CI 2.180–5.943, *P* < 0.001) or multivariate COX regression analysis (HR 3.566, 95% CI 2.118–6.004, *P* < 0.001), after adjusting other clinical features such as age, gender, tumor histological grade (grade) and TNM stage (T, N, M), RS was still an independent prognostic factor for GC patients.

### Validation of the angiogenesis-related prognostic model

**Fig. 5:**
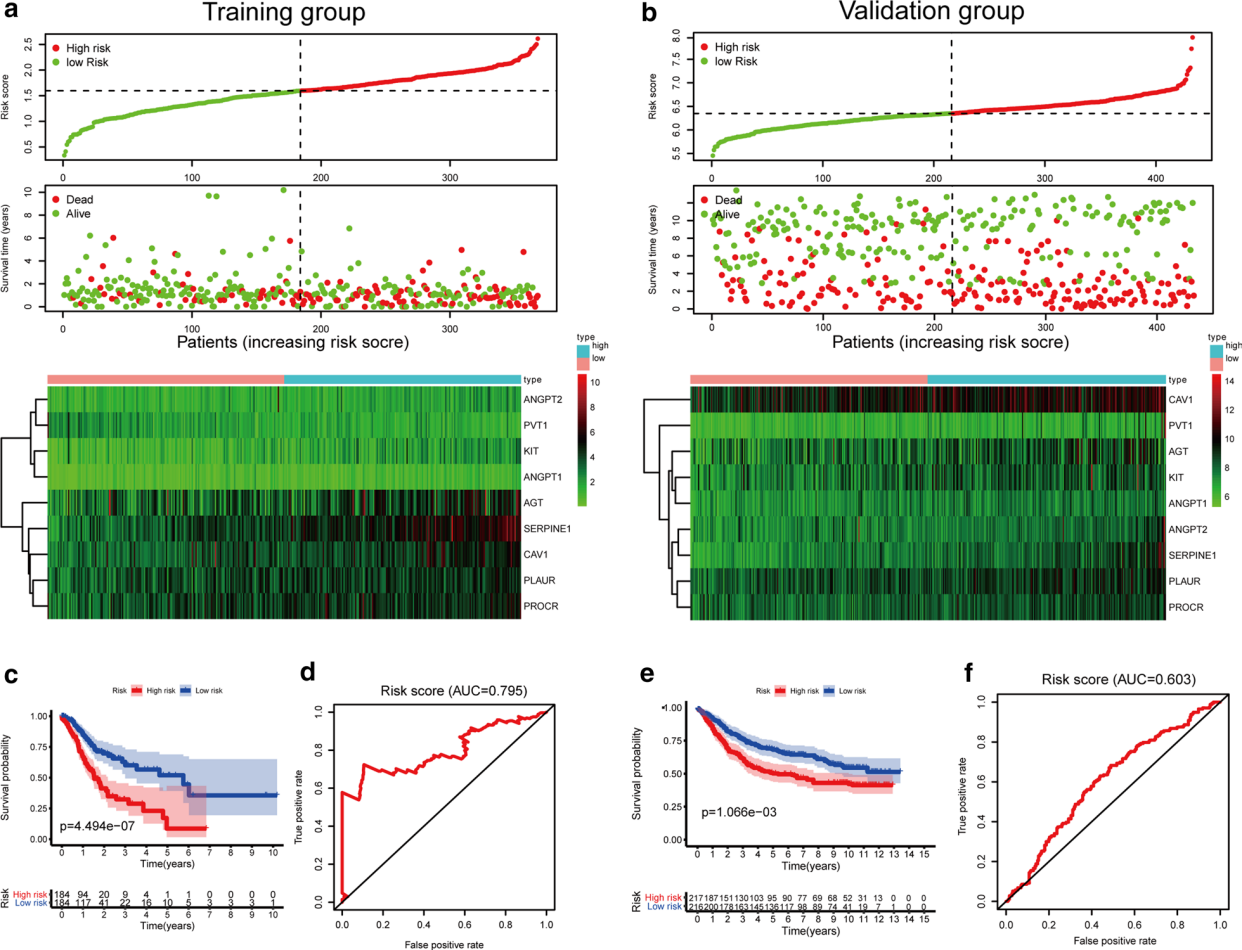
Development of RS based on the 9 ARGs signature of patients with GC in TCGA and GEO. **a**, **b** The RS distribution, vital status of patients and heatmap of the 9 ARGs expression profiles between high risk group and low risk group in training or validation group. **c**, **e** Kaplan–Meier analysis of the prognostic model in TCGA or GEO datasets. **d**, **f** Time-dependent ROC analysis showing the optimal AUC of the gene signature in the two cohorts

This angiogenesis-based prognostic signature could work as a predictive tool to evaluate the prognosis of patients with gastric cancer. In the training group from TCGA-STAD cohort, each patient will get a RS based on the expression of 9 ARGs. Taking the median RS in all patients as the cut-off value, the whole group was divided into high-risk group and low-risk group. There were significant differences in RS distribution, vital status of patients and heatmap of the 9 ARGs expression profiles between high risk group and low risk group (Fig. 5a). The OS in the low-risk group was significantly better than that in the high-risk group (*P* < 0.001) (Fig. 5c). ROC curve (AUC) analysis showed that RS has a considerable diagnostic and prognostic values on patients with GC (AUC = 0.795) (Fig. 5d). Then, we employed a validation group in GSE84437 cohort from GEO datasets with 433 GC cases and used OS to verify the effectiveness of the angiogenesis genes prognostic model. As expected, the validation group patients in the high-risk group distinguished by the prognostic model had a worse OS than those in the low-risk group (Fig. 5b, e). Moreover, the ROC curve shows the same results (Fig. 5f).

**Fig. 6:**
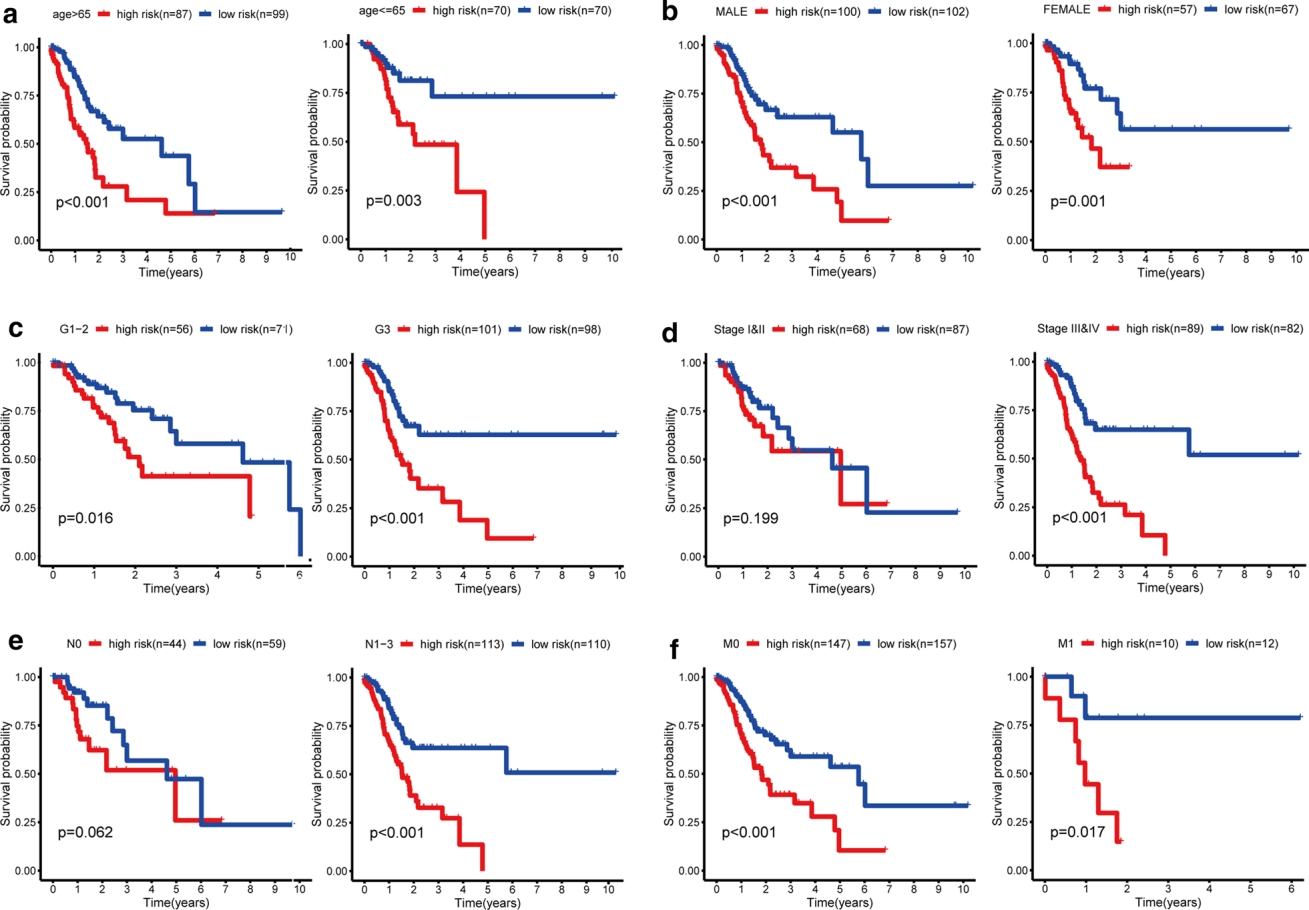
Stratified analysis of the relationship between RS score and survival rate of patients with gastric cancer in TCGA cohorts. **a** Age > 65 years and age ≤ 65 years. **b** female sex and male sex. **c** G1-2 and G3. **d** Stage I&II and stage II&III. **e** NO stage and N1-3 stage. (f) M0 stage and M1 stage

Then the patients in the training group were stratified according to the clinical characteristics, and the correlation between RS and OS in patients with GC was analyzed. The results showed that under the stratification of different clinical characteristics, RS had a good prognostic effect on patients with GC, especially in patients with advanced GC (Fig. 6a–f). In addition, we found that the expression of VEGFA was significantly up-regulated in tumor tissues (Additional file 1: Fig. 1a). However, in TCGA and GEO databases, the expression of VEGFA had no significant association with the prognosis of GC patients (Additional file 1: Fig. 1b and c).

### Construction of a nomogram

**Fig. 7:**
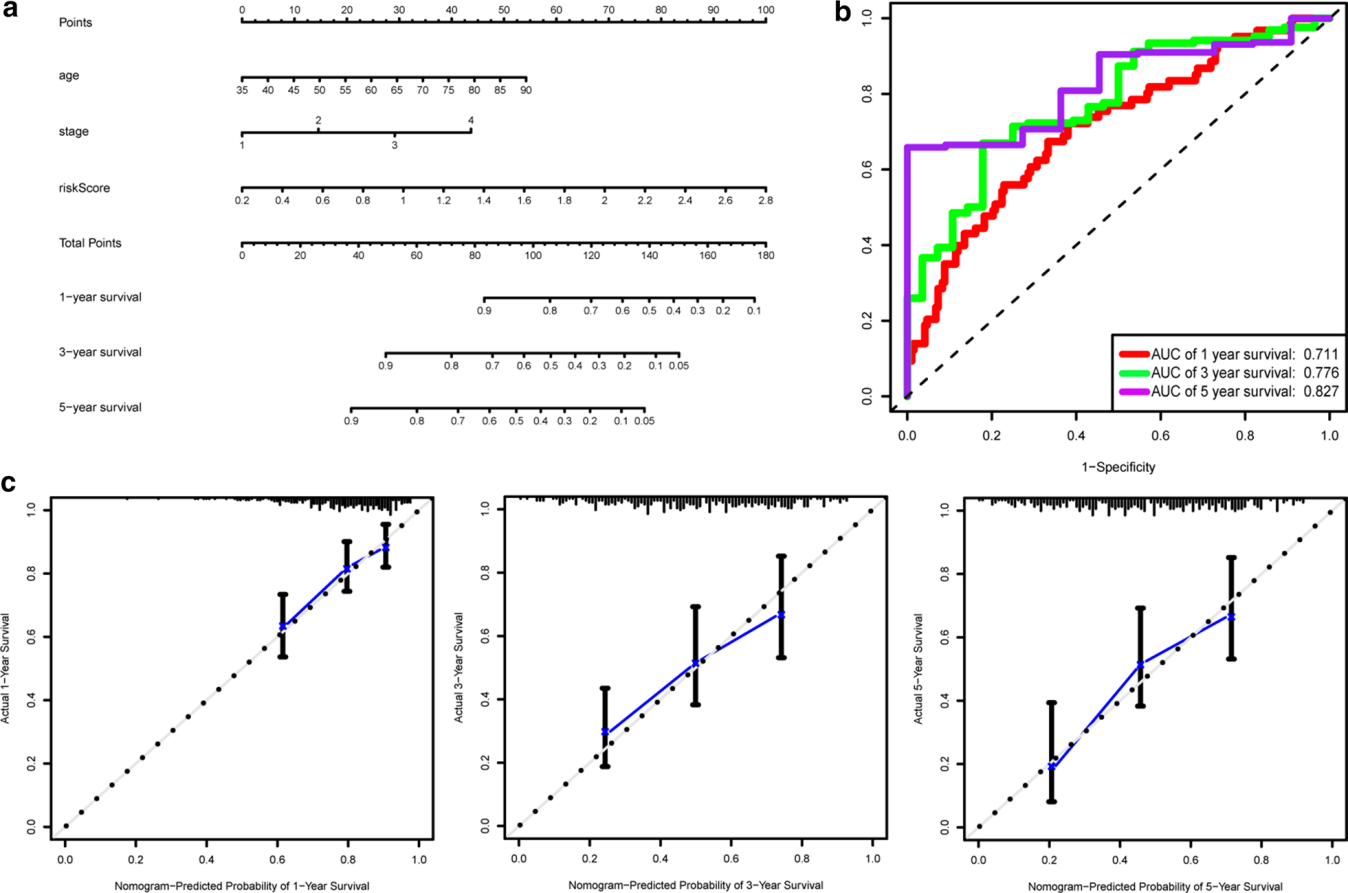
Nomogram for predicting of 1-, 3- and 5-year overall survival (OS) based on the nine ARGs signature. **a** A nomogram based on the risk scores, clinical stage and age of GC patients. **b** ROC analysis of the nomogram for predicting the 1-, 3- and 5-year OS. **c** Calibration curves of nomogram for survival prediction at 1-, 3- and 5-year

According to Cox regression combined with the significant clinical parameters, the parameters contains age, stage and risk score were selected to construct nomogram (Fig. 7a). Each patient will get a score according to the prognostic parameters, and the higher the total score indicates a worse outcome. Moreover, the ROC curves of 1-, 3- and 5-year OS indicated that our model has good predictive ability (Fig. 7b). Calibration plots had demonstrated a great consistency between the predicted and observed outcomes (Fig. 7c).

## Discussion

Gastric cancer is considered to be one of the most malignant tumors in the world due to high recurrence rate and low survival rate [18]. For clinicians, it is still challenging to predict the prognosis and risk stratification of patients with gastric cancer. Due to the limitations of TNM staging system and other scoring systems, there is an urgent need for new molecular biomarkers to predict the survival of patients with GC [19]. In the current study, we constructed and validated a gastric cancer risk model based on ARGs. As far as we know, this is the first study to explore prognostic biomarkers in patients with GC using angiogenesis-related gene scoring model.

Angiogenesis is a physiological process during tissue repair and regeneration, such as reproduction, embryonic development and wound healing [20]. Under normal quiescent, endothelial cells can sense angiogenic signals and participate in angiogenesis by maintaining a high degree of plasticity under controlled conditions. However, in many disease states, such as cancer, rheumatoid arthritis and atherosclerosis, uncontrolled angiogenesis will further promote the development of the disease and become a hallmark of these disease states [21]. The growth and metastasis of gastric cancer also depend on angiogenesis. After being stimulated by hypoxia and energy deficiency, tumor cells and tumor microenvironment will release a large number of angiogenic factors to trigger angiogenesis. Then neovascularization will provide oxygen and energy to the tumor and further promote tumor growth. Angiogenic genes play an important role in tumor growth and prognosis.

With the wide application of high-throughput array, the combinatorial analysis of angiogenic genes involved in the carcinogenicity of GC has been realized [22]. In this study, we collected transcriptional expression data and corresponding clinical data from TCGA and GEO databases. Then the differential expression of ARGs between GC samples and nontumor samples were obtained. Finally, a prognostic model based on 9 prognosis-related ARGS (AGT, ANGPT1, SERPINE1, ANGPT2, PVT1, PROCR, KIT, PLAUR and CAV1) was constructed, showing a good prognostic value in TCGA and GEO databases.

These differentially expressed ARGs are mainly concentrated in P13K-Akt, Rap1 and MAPK pathways and involved in the angiogenesis of GC. PI3K/AKT is an important intracellular signal transduction molecule, which participates in the regulation of cell proliferation, apoptosis and differentiation, and can regulate the expression of VEGF and hypoxia inducible factor (HIF-1) by activating kinases p70S6K1 and HDM2 [23]. VEGF is the main regulatory factor involved in tumor angiogenesis, while hypoxia can stimulate the secretion of angiogenic factors. Besides, PI3K-Akt pathway also plays an important role in hematopoiesis and angiogenesis mediated by K-ras signal pathway [24]. MAPK signal pathway is one of the important signal transduction systems in organisms, which plays an important role in cell survival, proliferation and angiogenesis [25]. A large number of studies have shown that RAP1 is activated in a variety of cancers, including leukemia and solid tumors [26, 27]. RAP1 plays a role in the invasion and metastasis of various tumor cells by regulating adhesion junctions and cytoskeleton remodeling.

Nine ARGs were used to establish the model equations for risk assessment. Among them, 4 candidate genes (ANGPT2, ANGPT1, PVT1, PROCR) were already reported to promote GC angiogenesis and tumorigenesis. ANGPT1 and ANGPT2 belong to the angiopoietin family, which play a central role in angiogenesis and are highly expressed in a variety of tumors such as GC, breast cancer and lung cancer [28–30]. Zhao et al. confirmed that PVT1 was upregulated and significantly associated with high-microvessel density and poor prognosis in GC [31]. Furthermore, overexpression of PVT1 in GC significantly increased the expression levels of angiogenesis-related transcription factors (STAT3, VEGFA, CTGF, ANGPT2) [32]. PROCR can promote tumor angiogenesis in vitro by activating ERK1/2 and AKT in GC cells, dependent on the activation of PAR1 [33].

It has already been proved that CAV1, SERPINE1 and AGT played important roles in the proliferation, migration and invasion of GC cells. What’s more, their expressions in tumors were candidate prognostic biomarkers for GC patients. Data from Gene Expression Profiling Interactive Analysis (GEPIA) revealed similar results. Wang et al. found that CAV-1 promoted drug resistance of GC cells through PI3K/Akt and MEK/ERK signaling pathways [34]. Another study indicated that positive Cav-1 expression in GC patients was associated with poor prognosis after radical gastrectomy [35, 36]. Plasminogen activator urokinase receptor (PLAUR) can be used as predictors of aggressive phenotypes in preoperative biopsies, Helicobacter pylori infection, and intestinal metaplasia [37]. The mutation of KIT is an important mechanism in gastrointestinal stromal tumors (GIST), but its role in GC is still unclear [38]. Further research on the roles of these genes in GC is still required.

So far, most of the tumor-related genes identified in bioinformatics methods were analyzed separately, which cannot fully reflect the process of tumorigenesis, and the role in the diagnosis and prognosis prediction is still poor. In addition, most studies focused on a single cancer, rather than on one specific type of cancer or one specific process in carcinogenesis. It will be more valuable to identify a cluster of genes with prognosis function in one specific cancer-related process. We generated a multigene signature in this study to predict the prognosis of individual GC patients, focusing on the tumor angiogenesis sets. However, this study also has some shortcomings. First of all, we checked the data in the public database, so the study could be more valuable if further experiments in GC cells and animal models are performed on these genes. Secondly, the validation group in this study is based on GEO database. The conclusions could be more powerful after being verified in a separate cohort.

In conclusion, our study confirmed the relevant genes and pathways in the angiogenesis process of GC. They could work as potential biomarkers to predict the prognosis and diagnosis of GC and provide new perspectives in targeted therapy.

## Supplementary Information

**Additional file 1: Supplementary Table I**. Baseline clinical characteristics of patients with gastric cancer in this research. **Supplementary Table 2**. The prognostic related ARGS screened by univariate Cox regression analysis. **Supplementary Fig. 1**. Differentially expression and prognosis of VEGFA in gastric cancer. (a) Differentially expressed VEGFA between GC and normal gastric tissues from TCGA cohort. (b) Kaplan–Meier analysis of the prognosis of VEGFA in TCGA cohort. (c) Kaplan–Meier analysis of the prognosis of VEGFA in GEO cohort.

## Data Availability

The datasets used and/or analyzed during the current study are available from TCGA repository: https://portal.gdc.cancer.gov/; GEO repository: https://www.ncbi.nlm.nih.gov/geo/; the GeneCards website: https://www.genecards.org/; the STRING database: http://www.string-db.org/; and the KEGG pathway database: www.kegg.jp/kegg/kegg1.html.
