# Supplementary material for: Identification of the angiogenesis related genes for predicting prognosis of patients with gastric cancer
Source: BMC Gastroenterol. 2021 Apr 1;21:146. doi: 10.1186/s12876-021-01734-4 (PMC8017607; doi:10.1186/s12876-021-01734-4)
Supplement: Supplementary file 1 — Additional file 1: Supplementary Table I. Baseline clinical characteristics of patients with gastric cancer in this research. Supplementary Table 2. The prognostic related ARGS screened by univariate Cox regression analysis. Supplementary Fig. 1. Differentially expression and prognosis of VEGFA in gastric cancer. (a) Differentially expressed VEGFA between GC and normal gastric tissues from TCGA cohort. (b) Kaplan–Meier analysis of the prognosis of VEGFA in TCGA cohort. (c) Kaplan–Meier analysis of the prognosis of VEGFA in GEO cohort. [file 12876_2021_1734_MOESM1_ESM.docx]

Identification of the angiogenesis related genes for predicting prognosis of patients with gastric cancer

Sheng Zheng ^1, 2*^, Zizhen Zhang ^1, 2*^, Ning Ding ^1, 2^, Jiawei Sun ^1, 2^, Yifeng Lin ^1, 2^, Jingyu Chen ^1, 2^, Jing Zhong ^1, 2^, Liming Shao ^1^, Zhenghua Lin^1, 2^, Meng Xue ^1, 2,^*

^1^ Department of Gastroenterology, the Second Affiliated Hospital of Zhejiang University School of Medicine, Hangzhou, China

^2^ Institute of Gastroenterology, Zhejiang University, Hangzhou, China

**Supplementary Table I.** Baseline clinical characteristics of patients with gastric cancer in this research.

| **Clinical characteristic** | **N** | **%** |
| --- | --- | --- |
| Tissue |  |  |
| Adjacent noncancerous tissue | 32 | 7.9 |
| Stomach adenocarcinoma | 375 | 92.1 |
| Age (years) |  |  |
| >65 | 207 | 55.7 |
| <=65 | 164 | 44.3 |
| Gender |  |  |
| Male | 240 | 64.0 |
| Female | 135 | 36.0 |
| T classification |  |  |
| T1-T2 | 100 | 27.2 |
| T3-T4 | 267 | 72.8 |
| N classification |  |  |
| N0-N1 | 207 | 58.0 |
| N2-N3 | 150 | 42.0 |
| M classification |  |  |
| M0 | 330 | 92.9 |
| M1 | 25 | 7.1 |
| Neoplasm histologic grade |  |  |
| G1 | 10 | 2.7 |
| G2 | 137 | 37.4 |
| G3 | 219 | 59.9 |
| UICC Stage |  |  |
| Stage I | 53 | 15.1 |
| Stage II-IV | 299 | 84.9 |

**Supplementary Table 2.** The prognostic related ARGS screened by univariate Cox regression analysis

| **Gene symbol** | **Hazard ratio** | **P value** |
| --- | --- | --- |
| F2R | 1.327(1.090−1.615) | 0.005 |
| CAV1 | 1.204(1.058−1.371) | 0.005 |
| PLAUR | 1.218(1.014−1.463) | 0.035 |
| COL4A1 | 1.265(1.038−1.540) | 0.020 |
| JAM3 | 1.232(1.036−1.464) | 0.018 |
| ADAMTS1 | 1.250(1.068−1.464) | 0.005 |
| KIT | 1.284(1.080−1.527) | 0.005 |
| CD36 | 1.290(1.055−1.577) | 0.013 |
| AGT | 1.154(1.034−1.289) | 0.011 |
| ANGPT1 | 1.472(1.065−2.034) | 0.019 |
| SERPINE1 | 1.250(1.113−1.403) | <0.001 |
| ANGPT2 | 1.267(1.022−1.572) | 0.031 |
| PDGFRB | 1.223(1.032−1.448) | 0.020 |
| PVT1 | 0.715(0.551−0.927) | 0.011 |
| SPARC | 1.284(1.093−1.508) | 0.002 |
| DCN | 1.172(1.029−1.334) | 0.017 |
| APLNR | 1.201(1.022−1.412) | 0.026 |
| PROCR | 1.213(1.028−1.430) | 0.022 |


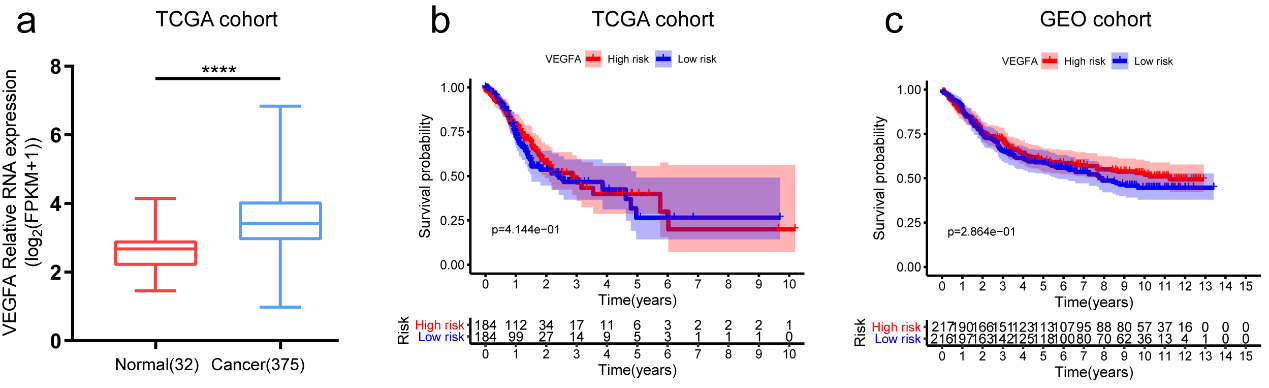


**Supplementary Fig.** 1 Differentially expression and prognosis of VEGFA in gastric cancer. (a) Differentially expressed VEGFA between GC and normal gastric tissues from TCGA cohort. (b) Kaplan–Meier analysis of the prognosis of VEGFA in TCGA cohort. (c) Kaplan–Meier analysis of the prognosis of VEGFA in GEO cohort.
